# Epoprostenol Delivered via High Flow Nasal Cannula for ICU Subjects with Severe Hypoxemia Comorbid with Pulmonary Hypertension or Right Heart Dysfunction

**DOI:** 10.3390/pharmaceutics11060281

**Published:** 2019-06-14

**Authors:** Jie Li, Lauren J. Harnois, Bethelhem Markos, Keith M. Roberts, Salma Al Homoud, Jing Liu, Sara Mirza, David Vines

**Affiliations:** Department of Cardiopulmonary Sciences, Division of Respiratory Care, Rush University Medical Center, Chicago, IL 60130, USA; Lauren_J_Harnois@rush.edu (L.J.H.); Bethelhem_Markos@rush.edu (B.M.); kmr8106@sbcglobal.net (K.M.R.); salmakamelh@gmail.com (S.A.H.); jane.liu.uru@gmail.com (J.L.); Sara_Mirza@rush.edu (S.M.); David_Vines@rush.edu (D.V.)

**Keywords:** high-flow nasal cannula, epoprostenol, inhalation, hypoxemia

## Abstract

Inhaled epoprostenol (iEPO) has been utilized to improve oxygenation in mechanically ventilated subjects with severe hypoxemia, but the evidence for iEPO via high-flow nasal cannula (HFNC) is rare. Following approval by the institutional review board, this retrospective cohort study evaluated subjects who received iEPO via HFNC for more than 30 min to treat severe hypoxemia comorbid with pulmonary hypertension or right heart dysfunction between July 2015 and April 2018. A total of 11 subjects were enrolled in the study of whom 4 were male (36.4%), age 57.5 ± 22.1 years, and APACHE II score at ICU admission was 18.5 ± 5.7. Ten subjects had more than three chronic heart or lung comorbidities; seven of them used home oxygen. After inhaling epoprostenol, subjects’ SpO_2_/F_I_O_2_ ratio improved from 107.5 ± 26.3 to 125.5 ± 31.6 (*p* = 0.026) within 30–60 min. Five subjects (45.5%) had SpO_2_/F_I_O_2_ improvement >20%, which was considered as a positive response. Heart rate, blood pressure, and respiratory rate were not significantly different. Seven subjects did not require intubation, and seven subjects were discharged home. This retrospective study demonstrated the feasibility of iEPO via HFNC in improving oxygenation. Careful titration of flow while evaluating subjects’ response may help identify responders and avoid delaying other interventions. This study supports the need for a larger prospective randomized control trial to further evaluate the efficacy of iEPO via HFNC in improving outcomes.

## 1. Introduction

Inhaled epoprostenol (iEPO) is a selective pulmonary vasodilator that has been shown to be equally effective as inhaled nitric oxide (iNO) in reducing pulmonary artery pressure [1,2,3,4] and improving oxygenation [5,6,7], with similar incidence of complications in mechanically ventilated subjects. Compared to systemic use of epoprostenol, iEPO has more specific pulmonary vasodilation effects. The reduction of pulmonary vasculature resistance was more significant while the systemic blood pressure was less affected [1]. Moreover, iEPO is 4.5 to 17 times less expensive than iNO, depending on the institution’s contracted price [3,7]. Due to these financial savings, there is a growing trend of substituting iEPO for iNO in mechanically ventilated subjects. However, the evidence for using iEPO in subjects who are extubated or spontaneously breathing is scarce. The only study using iEPO delivered via mask in spontaneously breathing subjects showed no differences between iEPO and iNO in regards to acute hemodynamic effects [4]. But However, iEPO delivery via mask is not feasible for long-term use due to its inconvenience and discomfort. Given the expense of using inhaled pulmonary vasodilators for pulmonary hypertension or hypoxemia subjects and the clinical impact of compliance with the prescribed therapy as well as the short half life time of iEPO, there is a need to determine a more feasible manner to deliver iEPO. 

An alternative to mask administration of iEPO for spontaneously breathing subjects is high-flow nasal cannula (HFNC), which has been shown to be effective in improving oxygenation and avoiding intubation in randomized controlled trials [8,9]. Consequently, HFNC is more suitable and feasible for long-term use. Aerosol delivery via HFNC may minimize interruptions and improve subject compliance [10]. HFNC has been shown to deliver clinically relevant masses of medicated aerosol in both in vitro [11,12,13,14] and in vivo studies [14,15]. Two retrospective studies in pediatric asthmatic and bronchiolitis subjects reported that combined use of vibrating mesh nebulizer (VMN) and heated HFNC to deliver bronchodilators helped avoid intubation [16,17]. Three recent clinical trials demonstrated that using a jet nebulizer [18] or VMN [19,20] to deliver bronchodilator via HFNC for chronic obstructive pulmonary disease (COPD) subjects has similar efficacy as jet nebulizer via mouthpiece. However, no clinical study has been completed to evaluate the efficacy of iEPO via HFNC or the dosage of iEPO needed via HFNC to produce a clinically significant effect.

Over the past three years, iEPO via HFNC has been used for subjects with hypoxemia comorbid with pulmonary hypertension or right heart dysfunction in the adult Intensive Care Units (ICUs) at Rush University Medical Center. This retrospective study was conducted to evaluate the impact of delivering iEPO via HFNC on oxygenation in spontaneously breathing subjects and assess its potential impact on subjects requiring mechanical ventilation. 

## 2. Materials and Methods 

### 2.1. Study Design 

Following approval by the institutional review board (No. 17062302-IRB01, approved on 8/8/2017 and amendment was approved on 11/19/2018) in Rush University Medical Center, a retrospective chart review was conducted in adult ICUs with a total 112 beds in a 644-bed tertiary university hospital. Subjects who were ≥18 years old and received iEPO via HFNC to treat severe hypoxemia were enrolled from a registry database in the respiratory care department. Severe hypoxemia was defined as requiring a fraction of inspired oxygen (F_I_O_2_) via HFNC >0.5 to maintain the blood oxygen saturation (SpO_2_) at 88–93%. Exclusion criteria were: (1) iEPO was utilized less than 30 min, (2) palliative care. 

### 2.2. iEPO Use via HFNC 

**Figure 1:**
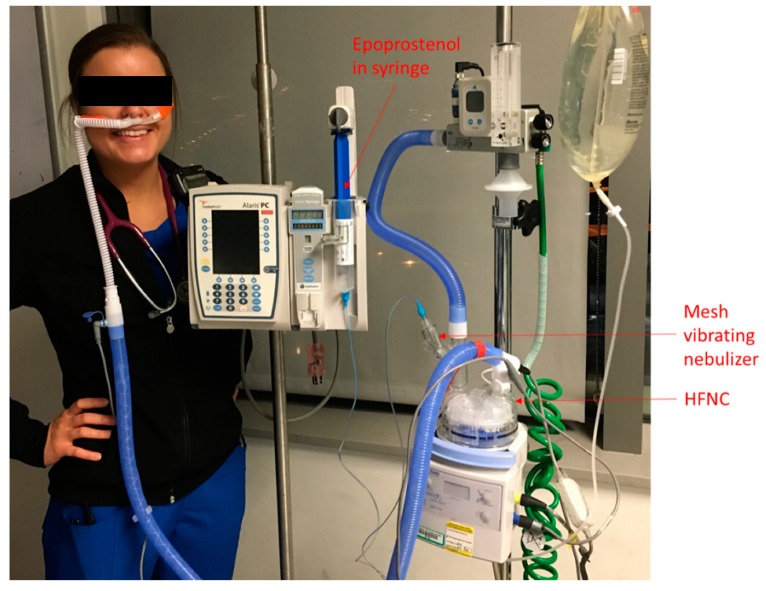
Set up for iEPO delivery via HFNC. iEPO, inhaled epoprostenol; HFNC, high-flow nasal cannula.

We started using iEPO in adult intensive care units in July 2015. A protocol was created to initiate iEPO which included step-by-step instructions, and the indications for using iEPO were as follows: (1) acute respiratory distress syndrome (ARDS) with partial pressure of oxygen in arterial blood/ fraction of inspired oxygen (PaO_2_/F_I_O_2_) ratio of ≤200, (2) Pulmonary hypertension with mean pulmonary arterial pressure (mPAP) ≥30 mmHg or systolic pulmonary arterial pressure (sPAP) ≥40 mmHg; (3) Right heart failure with central venous pressure (CVP) ≥15 mmHg with cardiac index (CI) <2.2 L/min/m^2^. When iEPO was delivered via HFNC, a vibrating mesh nebulizer (Aerogen ^®^ Solo, Aerogen Ltd., Chicago, USA ) was placed upstream (dry side) of a heated humidifier in the Optiflow^TM^ system (MR 850, Fisher & Paykel, Auckland, New Zealand) (Figure 1). Flow was initiated at 30 L/min and then titrated up to 50 L/min if subjects tolerated it. 

Veletri (epoprostenol) (1.5mg) was reconstituted with sterile water, according to the manufacturer’s instructions. The solution was drawn into a 60–mL syringe and attached to the VMN reservoir via a segment of tubing. The syringe was connected to the Alaris Syringe Module (Carefusion, San Diego, CA, USA), which was configured to administer continuous nebulization of epoprostenol. The dose of iEPO was decided by subjects’ ideal body weight starting at 50 ng/Kg/min and weaned by 10 ng/Kg/min. The duration of iEPO was determined based on the subject’s clinical response. 

### 2.3. Data Collection

Subjects’ demographic information including race, age, gender, medical history, and Acute Physiologic Assessment and Chronic Health Evaluation (APACHE II) score at ICU admission was collected. Heart rate, blood pressure, respiratory rate, SpO_2_, HFNC flow, and F_I_O_2_ were collected at 30–60 min before and 30–60 min after iEPO initiation. 

The primary outcome was oxygenation improvement. Because of the retrospective nature, arterial blood gases were not always available before and after initiation of iEPO, so SpO_2_/F_I_O_2_ was substituted for PaO_2_/F_I_O_2_ to evaluate subjects’ oxygenation [21,22,23]. A subject was considered a responder to iEPO if their SpO_2_/F_I_O_2_ increased by 20% or more. Secondary outcomes were the incidence of intubation, complications including systemic hypotension, ICU stay, HFNC duration, duration of iEPO, and ICU survival. 

Vasopressors (included the type and dose) and extracorporeal membrane oxygenation (ECMO) (if applicable) settings 30–60 min pre and post iEPO were also reviewed. If any change of the type and dose on the vasopressors or ECMO setting was made, the change would be collected. 

### 2.4. Statistical Analysis

The Kolmogorov–Smirnov statistic tests were used to test the normality of distribution for considered variables. Continuous variables (pre and post iEPO) were expressed as mean (standard deviation [SD]) or median (Inter-Quartile Range [IQR]) and compared with Wilcoxon sign rank test, whereas differences in categorical variables were assessed with the chi-square test. A *p*-value of <0.05 was considered to be statistically significant for all tests. Data analysis was conducted with SPSS statistical software (SPSS 23.0 for windows; SPSS; Chicago, IL, USA).

## 3. Results

### 3.1. Demographic Information

Between July 2015 and April 2018, 21 subjects received iEPO via HFNC, 10 subjects were excluded, and 11 subjects were enrolled. The reasons for exclusion were: 4 subjects were using iEPO during invasive ventilation and extubated to HFNC with iEPO for treating pulmonary hypertension, 4 subjects whose PaO_2_/ F_I_O_2_ > 300 used HFNC and iEPO for pulmonary hypertension; 1 subject used iEPO less than 10 min just for a pulmonary vaso-reactivity test; 1 subject was extubated to HFNC and iEPO for palliative care. 

Among the 11 enrolled subjects, 4 (36.4%) were male, with a mean (±SD) age of 57.5 ± 22.1 yrs. The APACHE II score at ICU admission was 18.5 ± 5.7. Five (45.5%) subjects were Caucasian, 5 (45.5%) were African American, and 1 (9.1%) was Hispanic. Ten subjects had chronic heart or lung comorbidities, and 7 of them used home oxygen at 2–6 L/min (Table 1). Six subjects had pulmonary hypertension, 2 subjects had right heart failure, and 3 subjects had both pulmonary hypertension and right heart failure. 

Only one subject started iEPO with HFNC simultaneously but did not respond to iEPO and HFNC (case 6 in Table 1). The other 10 subjects were placed on HFNC for 21 (1, 44) hours with flow setting at 40 (30, 50) L/min prior to starting iEPO. The dose of iEPO was initiated at 50ng/Kg/min in all except one subject (case 5 in Table 1), who started at 20 ng/Kg/min and was on veno-venous extracorporeal membrane oxygenation (VV-ECMO). One other subject was also on VV-ECMO while iEPO and HFNC were initiated. 

### 3.2. Oxygenation Effects and Safety

**Figure 2:**
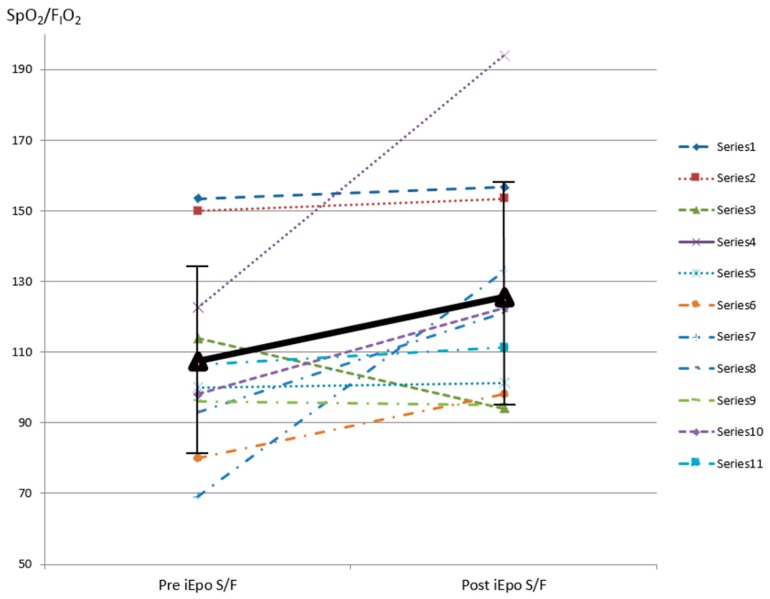
SpO_2_/F_I_O_2_ pre and post iEPO via HFNC was initiated. SpO_2_ pulse oximetry saturation; F_I_O_2_ fraction of inspired oxygen; iEPO inhaled epoprostenol; HFNC high-flow nasal cannula.

After inhaling epoprostenol, subjects’ SpO_2_/F_I_O_2_ improved from 107.5 ± 26.3 to 125.5 ± 31.6 within 30–60 min (*p* = 0.026) (Figure 2). Five (45.5%) subjects had a SpO_2_/F_I_O_2_ improvement greater than 20%. Two of these subjects’ SpO_2_ increased from 69% (case 8) and 80% (case 3) on F_I_O_2_ 1.0 to 93% on F_I_O_2_ 0.7 and 98% on F_I_O_2_ 1.0, respectively. When comparing these five responders with the six non-responders, responders’ SpO_2_ improved from (87.6% ± 12.7%) to (96.6% ± 2.1%), which was significantly higher than post-iEPO SpO_2_ among non-responders (*p* = 0.03) (Table 2). There was no difference of comorbid pulmonary hypertension or right heart failure, HFNC flow while iEPO was being delivered, intubation, hospital survival, ICU, and hospital stay between responders and non-responders.

Heart rate, blood pressure, and respiratory rate of pre and post iEPO were not significantly different (Table 3). None of the vasopressors or ECMO settings was changed within 30–60 min of iEPO initiation. None of the subjects had to discontinue iEPO and HFNC due to discomfort or intolerance.

### 3.3. Outcome 

Seven subjects including both responders and non-responders to iEPO did not require intubation, and ultimately 7 subjects were discharged from the hospital with 12 (9, 22.5) days of ICU stay. Two subjects were previously on VV-ECMO when iEPO via HFNC was initiated, and 1 withdrew therapy. 

## 4. Discussion

In our study, we found that subjects’ oxygenation was improved after iEPO was initiated via HFNC, which was consistent with the iEPO effects on the mechanically ventilated patients [5,6,7] and spontaneous breathing patients with HFNC in Ammar et al.’s study [24]. However, Ammar et al. did not differentiate the effects of HFNC and iEPO on oxygenation improvement. HFNC has been shown to improve oxygenation, thus it is difficult to isolate the benefits of iEPO from HFNC [25]. In our study, only 1 subject started iEPO and HFNC simultaneously, but did not respond. The other 10 subjects had utilized HFNC for ≥1 h, which was a sufficient time interval for SpO_2_ to have stabilized from the increase in delivered F_I_O_2_ attributed to HFNC. Additionally, the HFNC flow setting was maintained or reduced after initiating iEPO in all patients, which should not have resulted in further improvement in oxygenation in the relative short period of time between the pre and post assessment of iEPO (30–60 min). Thus, the improvement of oxygenation most likely was due to the initiation of iEPO. Moreover, no complication, such as systemic hypotension, was observed in the subjects after the initiation of iEPO via HFNC. With this novel route, intubation may have been avoided in a majority of the subjects. In two of these subjects (cases 3 and 8), iEPO was utilized as the last resort before intubation.

It should also be acknowledged that not all of the hypoxemia subjects responded to iEPO. Identifying responders to iEPO might help discern patients likely to benefit from this combination therapy and avoid delaying other interventions such as intubation [26]. In Kallet et al.’s study on mechanical ventilation, 208 ARDS subjects were included, and only 62% responded to iEPO. The response rate decreased if the primary source of ARDS was lung related [27]. This finding might explain the low response rate (45%) in our study as all of the subjects had pulmonary etiology. The other explanation might come from subjects’ baseline oxygenation. Kallet et al. found that the baseline oxygenation was significantly related to ARDS patients’ response to iEPO while patients with lower baseline oxygenation had less response to iEPO [27]. In our study, all the subjects’ baseline SpO_2_/F_I_O_2_ was ≤150, which was in the lowest group in Kallet et al.’s study. As a practical substitute to PaO_2_/ F_I_O_2_, SpO_2_/F_I_O_2_ has been shown to have a strong linear relationship in moderate to severe ARDS [22,23] and was recommended as a diagnostic tool for early enrollment in clinical trial [23].

Flow has been reported to be a critical factor in the lung deposition of aerosol delivery via HFNC [11,12,13,14]. If flow was increased from 30 to 50 L/min in quiet breathing subjects, the lung deposition was found to decrease from (11.6 ± 1.2) % to (3.5 ± 0.2) % in a bench study [12] and from (3.76 ± 1.36) % to (2.23 ± 0.81) % in a scintigraphy study [14]. However, Réminiac et al. [11] and Dailey et al. [12] found that distressed breathing significantly increased lung deposition. Our previous adult and pediatric in vitro studies found that inhaled dose was higher with gas flow below subjects’ inspiratory flow than that with gas flow exceeding subjects’ inspiratory flow [13,28], with optimal gas flow rate at 50% patient’s inspiratory flow [28]. Subjects in our study had tachypnea, so the optimal flow for them may be higher in order to generate better aerosol deposition than the gas flow for the healthy volunteers in the scintigraphy study. Subjects receiving a higher lung dose may have been responders to iEPO since response is dose related [5]. More prospective studies are needed to further explore the relationship between lung dose and flow on subjects’ response in hypoxemic patients as well as to explore the effective dose of iEPO via HFNC. 

Besides the benefits from high flow in aerosol delivery to hypoxemic patients, the positive expiratory pressure (PEP) also influences the lung deposition of aerosol delivery, which might be due to the increment of end-expiratory lung volumes. Alcoforado et al. found that the combination of a PEP device and jet nebulizer had significantly higher lung deposition than the jet nebulizer alone [29]. HFNC generates 2–4 cmH_2_O positive expiratory pressure for adults on higher flow rate during HFNC when the subject’s mouth is closed [30]. Therefore, higher flow not only meets hypoxemia patients’ inspiratory flow demand, but may optimize aerosol delivery. This combination of HFNC with a high flow rate and iEPO may be beneficial for adult subjects who have severe hypoxemia with pulmonary hypertension or right heart dysfunction.

Pacheco et al. found that ARDS patients’ response to iEPO was identified as an independent predictor of 90-day mortality [31]. Kallet et al. also found that patients who responded to iEPO had an improved hospital and 90-day outcome [26]. These findings imply that closely monitoring patients’ response to iEPO may provide important feedback regarding their prognosis. Due to our small sample size, the mortality difference observed between groups cannot be extrapolated to demonstrate an overall benefit. Future studies on ARDS patients’ response to iEPO via HFNC with larger sample size are necessary to explore these findings. 

A major limitation of this study was its retrospective nature. Due to this, we were unable to evaluate subjects’ pulmonary vasculature resistance or heart function as only two subjects had a pulmonary artery catheter placed, and neither of them had the parameters measured 30–60 min before and after iEPO was initiated. ICU patients’ situation changed fast, thus immediate assessment might better reflect the direct response from iEPO. However, acquiring ABG 30–60 min before and after initiating iEPO for all patients was not realistic, thus SpO_2_/F_I_O_2_ had to be substituted for PaO_2_/F_I_O_2_ as a means for evaluating oxygenation [21,22,23]. Additionally, if the HFNC flow was set lower than subjects’ inspiratory flow, the actual F_I_O_2_ subjects inhaled was lower than the setting values due to the air entrainment. However, HFNC flows for all the subjects in our study were maintained or reduced after iEPO was initiated, thus using SpO_2_/F_I_O_2_ to evaluate subjects’ response was stricter, as the actual F_I_O_2_ post-iEPO might be lower than the setting value which was utilized to calculate. A second limitation was the small sample size. This could explain why the difference in responders and non-responders was not significant, except for SpO_2_/F_I_O_2_. A larger sample size in a prospective pre-post study is needed. Thirdly, we did not have the control group to compare the difference of HFNC with or without iEPO, especially the long-term benefits and outcomes. The results from this study need to be interpreted cautiously, and a randomized control trial is needed.

## 5. Conclusion

This retrospective study demonstrated the feasibility of inhaled epoprostenol via HFNC in improving oxygenation in adult subjects with severe hypoxemia with pulmonary hypertension or right heart dysfunction. Carefully titrating flow and cautiously evaluating clinical response may help identify responders and avoid delaying other interventions. Overall, this study supports the need for larger prospective randomized control trials to further evaluate the efficacy of iEPO via HFNC.

## Figures and Tables

**Table 1:** Individual subject’s demographic characteristics, response to iEPO and outcome.

ID	Gender	Race*	Age	APACHE II	Diagnosis ^#^	Home O_2_	HFNC Hours Pre- iEPO	Pre- iEPO SpO_2_	Pre- iEPO FiO_2_	Pre- iEPO Flow	Post- iEPO SpO_2_	Post- iEPO FiO_2_	Post- iEPO Flow	iEPO Duration (Mins)	Respond ^&^	Intubation	Outcome
1	M	2	85	26	HTN, HFpEF, PH, COPD, CVA, PVD, CKD, BPH s/p TURP	Yes	11.5	92	6	40	94	6	40	176	No	No	Died in hospice
2	F	1	69	18	DLBCL s/p chemo, acute respiratory failure with hypoxia, sepsis; PAH	No	44	96	1.0	50	95	1.0	40	1135	No	Yes	Alive
3	F	3	23	7	Myocarditis due to influenza A virus, PH	No	28	80	1.0	50	98	1.0	50	6276	Yes	No	Alive
4	F	1	72	22	HTN, breast cancer, and IIIA non-small cell lung cancer s/p lobectomy; septic shock; RHF	No	82	98	1.0	45	98	8	45	13662	Yes	Yes	Alive. VV-ECMO with iEPO
5	M	1	51	27	CAD, COPD, stage III squamous cell carcinoma of lung; RHF from PE	No	21	90	9	40	91	9	40	1400	No	No	Died. VV-ECMO with iEPO,
6	F	2	42	14	SLE, HTN, CHF, CAD, CKD, ILD, PH	Yes	0	90	6	/	92	6	30	1298	No	No	Alive
7	F	1	61	16	group 1 PAH with connective tissue disease	Yes	1	91	8	35	94	1.0	35	4104	No	No	Alive.
8	F	2	23	14	ASD; biventricular systolic dysfunction; PH (WHO group 1 2/2 ASD with Eisenmenger’s), chronic hypoxemia	Yes	30.5	69	1.0	20	93	7	20	2089	Yes	No	Alive
9	M	1	78	19	CHF, PAH (group 1 functional class 3), ASD.	Yes	2	93	1.0	50	97	8	45	3184	Yes	No	Alive
10	M	2	71	21	PH (moderate R to L shunt), HFrEF (EF 35–40%) c/b VF s/p ICD, hypertension, CKD stage IV, DM c/b retinopathy, glaucoma, HLD	Yes	1	98	8	30	97	5	30	944	Yes	Yes	Died, withdrew therapy
11	F	2	63	19	PH (chronic), chronic hypoxemic respiratory failure, atypical carcinoid lung tumor, DM II, CKD, CAD, asthma	Yes	54	85	8	30	89	8	30	9921	No	Yes	Died

* 1 Caucasian; 2 African American; 3 Hispanic; ^#^ HTN: hypertension; HFpEF: heart failure with preserved ejection fraction; PH: pulmonary hypertension; COPD: chronic obstructive pulmonary disease; CVA: cerebrovascular accident; PVD: peripheral vascular disease; BPH s/p TURP: benign prostatic hyperplasia status post transurethral resection of the prostate; DLBCL: diffuse large B-cell lymphoma; PAH: pulmonary arteria hypertension; RHF: right heart failure; CAD: coronary artery disease; PE: pulmonary embolism; SLE: systemic lupus erythematosus; CHF: congestive heart failure; CKD: chronic kidney disease; ILD: interstitial lung disease; ASD: atrial septal defect; HFrEF: heart failure with reduced ejection fraction; VF: ventricular fibrillation; ICD: implantable cardioverter defibrillator; DM: diabetes mellitus; HLD: hypersensitivity lung disease; ^&^ A subject was considered a responder to iEPO if their SpO_2_/FIO_2_ increased by >20%.

**Table 2:** Comparison between responders and non-responders.

Items	Responders	Non-Responders	*p*
Number of subjects	5	6	
Pulmonary hypertension	4	5	/
Right heart failure	3	2	0.567
Pre respiratory rate	25.8 ± 9.4	25.8 ± 7.8	0.931
Pre F_I_O_2_	96 ± 8.9	78.3± 16.0	0.082
Pre flow	39 ± 13.4	35.8 ± 4.9	0.456
Pre SpO_2_	87.6 ± 12.7	90.7 ± 3.6	0.792
Pre SpO_2_/F_I_O_2_	92.5 ± 20.2	120 ± 25.3	0.082
Post respiratory rate	26.4 ± 4.9	26.3 ± 8.8	0.931
Post F_I_O_2_	76 ± 18.2	81.7 ± 18.3	0.662
Post flow	38 ± 12.6	35.8 ± 4.9	0.537
Post SpO_2_	96.6 ± 2.1	92.5 ± 2.3	0.03
Post SpO_2_/F_I_O_2_	133.7 ± 36	118.6 ± 28.9	0.429
Intubation	2	3	/
ICU stay	12 (7, 24)	14 (10, 21)	0.931
Hospital stay	12 (7, 24)	16 (11, 21)	0.792
Hospital survival	4	3	/

**Table 3:** Comparison between pre and post iEPO.

Items	Pre iEpo	Post iEpo	*p*
HR (beats/min)	108.1 ± 31.1	101.1 ± 20.4	0.284
mBP (mmHg)	87.7 ± 15.8	89.4 ± 16.2	0.824
RR (breaths/min)	25.8 ± 8.1	26.4 ± 7.0	0.681
SpO_2_ /F_I_O_2_	107.5 ± 26.3	125.5 ± 31.6	0.026
F_I_O_2_	0.9 (0.8, 1.0)	0.8 (0.65, 0.95)	0.129
SpO_2_ (%)	91.5 (90, 96)	94.4 ± 3.0	0.016
Flow (L/min)	40 (30, 50)	40 (30,45)	0.066
